# Development of Dermal Films Containing Miconazole Nitrate

**DOI:** 10.3390/molecules23071640

**Published:** 2018-07-05

**Authors:** Magdalena Bîrsan, Mihai Apostu, Nicoleta Todoran, Paula Antonoaea, Aura Rusu, Adriana Ciurba

**Affiliations:** 1 Faculty of Pharmacy, “Grigore T. Popa”, University of Medicine and Pharmacy of Iași, Iași 700115, Romania; magdalena.birsan@umfiasi.ro (M.B.); mihai.apostu@umfiasi.ro (M.A.); 2 Faculty of Pharmacy, University of Medicine and Pharmacy of Tîrgu Mureș, Târgu Mureş 540001, Romania; paula.antonoaea@umftgm.ro (P.A.); aura.rusu@umftgm.ro (A.R.); adriana.ciurba@umftgm.ro (A.C.)

**Keywords:** miconazole nitrate, hydroxypropyl methylcellulose, hydroxyethyl cellulose, Franz cell, dermal films

## Abstract

This study aims to develop new antifungal dermal films based on their mechanical properties (elongation, adhesion, behaviour towards vapour moisture) and the in vitro availability of miconazole nitrate, used as a pharmaceutical active ingredient in various concentrations. The three polymeric films prepared were translucent or shiny, with the surface of 63.585 cm^2^, 0.20–0.30 mm thickness, and content of miconazole nitrate of 3.931 or 15.726 mg·cm^2^. The mechanical resistance and elongation tests demonstrated that the two films based on hydroxyethyl cellulose (HEC) polymer were more elastic than the one prepared with hydroxypropyl methylcellulose (HPMC). The vapour water absorption and vapour water loss capacity of the films revealed that the HPMC film did not dry very well in the process of preparation by the evaporation of the solvent technique, unlike the HEC films that jellified more evenly in water and had higher drying capacity at 40 °C. The in vitro availability of miconazole nitrate from dermal films was evaluated using the Franz diffusion cell method, through a synthetic membrane (Ø 25 mm × 0.45 µm) and acceptor media with pH 7.4 (phosphate buffer and sodium lauryl sulphate 0.045%), resulting a release rate of up to 70%.

## 1. Introduction

The development of pharmaceutical forms begins with the preformulation process, which establishes the physical and chemical, pharmaceutical and technological, pharmacological and toxicological features of substances [1]. As an active pharmaceutical ingredient, miconazole nitrate (MN) is a broad spectrum antifungal agent which has already been studied in the form of mucoadhesive tablets, polymeric matrices (chitosan, gelatine, gum arabic, alginate, carbopol or acrylic resins), microgels, nano-lipid gels or nano-vesicles formulated to treat oral or vaginal candidiasis [2,3,4,5,6,7]. In addition to its antifungal activity, MN also has an antimicrobial action—the imidazole class, which makes it frequently applied on skin mucosa to heal fungal infections [8]. It inhibits the synthesis of ergosterol, a key component of fungal cell membranes [9]. For external use, MN is characterized by very good action as it easily penetrates the skin, and because of its efficient concentration the therapeutic effects last more than four days from the time of application [10,11]. Therefore, the development of MN-dermal films can bring various benefits to patients resistant to classical antifungal pharmaceutical forms because this active substance lasts longer in the skin layers, acting as a slow release product. Repeated use multiplies the dose in the corneous layer, leading to the disappearance of the mycosis infection. Initially, it can be started with a smaller dose and afterwards tripled depending of the type of the mycosis infection [12,13]. Dermal films represent in medical practice an alternative for the administration of certain drugs (other than the common oral, or intravenous and intramuscular administration methods) [14,15,16,17,18,19,20,21]. They are generally formulated with pressure sensitive adhesives that ensure the products adherence to the skin [22].

The polymers used as film formers are particularly important because between them and the bioadhesivity of the resulting film there is a directly proportional relation. Cellulose derivatives, such as hydroxypropyl methylcellulose (HPCM) and hydroxyethyl cellulose (HEC), are semisynthetic polymers with pronounced jellification capacity in water and consequently with the potential ability to form adhesive films, both on skin and mucosa. By their non-ionic character they provide stability, especially for ionizable molecules, thus being suitable to include salts (e.g., MN) in polymer-based matrices. Unlike other polymers, cellulose derivatives are generally dissolved by heating to form dispersions whose viscosity are not influenced by pH and in which they form gels by cooling or by solvent removal (due to the increase in polymer concentration) [23]. HEC is frequently used in the formulation of gels with mucous administration because it was demonstrated to be similar to the fertile cervical mucus [24,25]. On the other hand, HPMC is used as excipient in more than 80% of the pharmaceutical products with oral administration [26] and, unlike HEC, has the property of retaining water, structuring the gel in a particular manner without dehydrating the biological substrate when it gets in contact with [27,28], thus providing comfort feel in occlusive state of application. The flexibility and the elasticity of the polymeric films are ensured by plasticizers (e.g., polyethylene glycol, propylene glycol and polysorbates) which often can also act as permeation activators, when they breach into the corneous layer of the epidermis [29,30,31,32,33,34,35,36,37].

This study aims to develop new antifungal dermal films based on their mechanical properties (elongation, adhesion, behaviour towards vapour moisture) and the in vitro availability of miconazole nitrate used as pharmaceutical active ingredient in two variants of concentrations, prepared in the form of two types of polymeric film matrices: HPMC and HEC.

## 2. Results and Discussion

### 2.1. Products Obtained in Form of Polymeric Matrix Films

Compositions used for the preparation of the studied dermal films by casting and solvent evaporation technique are shown in Table 1, and the physical characteristics of the resulting dermal films, as well as the calculated content of miconazole nitrate, are shown in Table 2.

### 2.2. Mechanical Properties of MN Dermal Films

#### 2.2.1. Tensile Strength and Elongation Capacity

**Figure 1:**
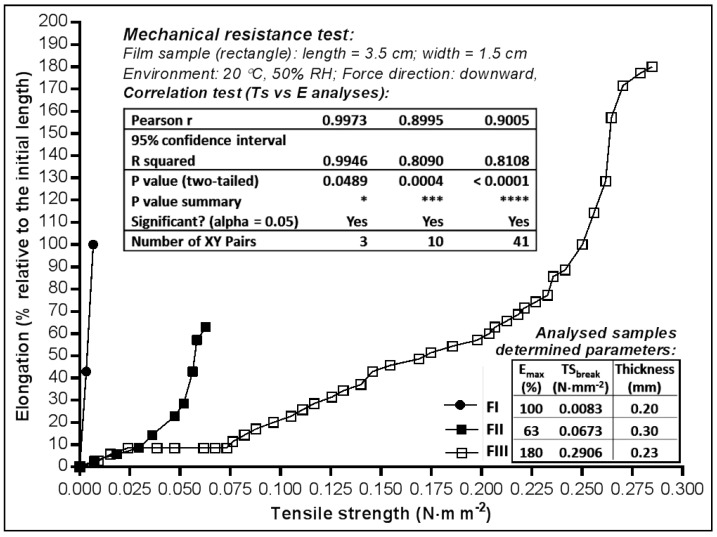
Elongation of dermal films as function of tensile strength.

The mechanical strength of dermal films (FI-FIII) was evaluated by testing their elongation capacity (E) under the action of descending traction forces, of increased intensity (Figure 1). The tension that led to the rupture of the film (TS_break_) had the smallest value for the HPMC—based film (FI—0.0083 N·mm^−2^) and the highest for the film prepared with 2% HEC (FIII—0.2906 N·mm^−2^). At a concentration of 3% HEC, the film resistance to breaking decreased by approximately 4× (FII—0.0673 N·mm^−2^). Such a behaviour indicates that the plasticizer (polyethylene glycol 400 (PEG_400_)) in concentration of 1% is insufficient to elasticize the structure of the polymeric matrix; this is also suggested by the maximum values of elasticity (E_max_—elongation recorded due to the tension applied before the film breaking), where the size relation was maintained (FII 63% and FIII 180%).

The value of E_max_ determined in the case of the HPCM film (Figure 1) does not in fact indicate the elongation capacity, but rather the plasticity level correlated to the low cohesion in the film mass, its elongation being determined almost exclusively by the value of the applied tension (Pearson r—0.9973), the film breaking at a tension up to 8–35× times (respectively) smaller compared to the films with HEC (FII, FIII). In these latter cases, HEC elasticizes the film almost to the same extent as it did when having a 2% concentration (Pearson r FIII—0.9005) or 3% (Pearson r FII—0.8995). Considering the mechanical strength and elongation, the most appropriate HEC concentration used at films preparation was that of 2%.

The results of FI were not as good as those of FII and FIII, fact that can be attributed to HPMC matrix which retained too much water, with a negative influence on elongation and mechanical strength. Although FI also contains other excipients for improving elongation and strength features, the HEC polymer matrix was demonstrated to be more elastic.

#### 2.2.2. Adhesive Capacity and Behaviour towards Vapour Moisture

**Figure 2:**
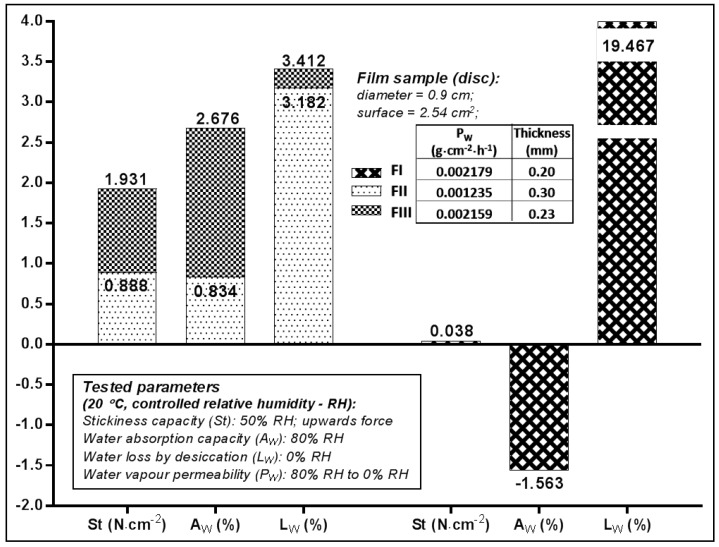
Properties of the film samples used for the in vitro Franz test.

The adhesive capacity of the films was determined and expressed by the value of the vertical ascendant force (S_t_), which caused a smooth and rigid surface to detach from the film surface. The values recorded at environmental temperature and humidity (Figure 2) indicated that FIII (S_t_ = 1.93 N·mm^−2^) had twice the adhesive capacity of FII, which suggests that 1% PEG_400_ was more suitable for HEC 2% concentration than 3% concentration.

The water vapours absorption (A_w_) caused by the very humid atmosphere (80% RH) was favoured by the use in the formulation of 2% HEC (A_w_ FIII—2.67%), compared to 3% HEC (A_w_ FII—0.83%). In the case of the film prepared with 1% HPMC (FIII) both the water vapours absorption capacity test (A_w_ of—1.56%), and the amount of water lost as vapours by desiccation (L_w_ of 19.46%) suggests this polymer, given its property to withhold water without dehydrating the contact surface—which could be skin or mucosa, provided the film a certain humidity since it still had a high content of volatile constituents.

The volatile content was much reduced—by approximately 3%—for the films prepared with HEC (FII, FIII) which suggests the preparation method and the formulation were more suitable for HEC as film former. The water vapours permeability (P_w_) of films ranged between 0.0012 and 0.0021 g·cm^−2^·h^−1^, FII (with 2% HEC) manifesting an occlusive effect of double intensity with respect to the other two (0.0012 vs. 0.0021).

### 2.3. In Vitro Availability of Miconazole Nitrate Dermal Films

#### 2.3.1. Analysis of In Vitro Release Profiles

**Figure 3:**
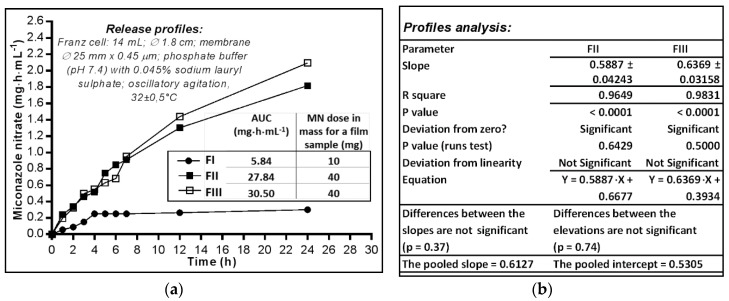
(**a**) The in vitro availability of miconazole nitrate dermal films expressed by AUC (area under curve); (**b**) Parameters calculated by linear regression of the released profiles (mg·cm^−2^ vs. h).

The ability of MN to be released from the polymeric matrix of the films was evaluated using the Franz cell diffusion method (Figure 3a). The HPMC film (FI) profile shows that after 3–4 h a plateau appeared that lasted up to 24 h; this behaviour could be attributed to the structural disintegration of the film under the moisture action. The amount of MN almost constantly released (approximately 30% of the film dose) seems more likely determined by the dissolution process—MN having a moderate solubility in water (26.3 µg·mL^−1^ in buffer at pH 7.4) but also positively influenced by the sodium lauryl sulphate included in the aqueous media used as acceptor compartment, rather than the diffusion of the substance among the macromolecules of the polymer that relaxes and swells [38] in the absorbed water.

On the other hand, the two films prepared with HEC 3% (FII) and 2% (FIII), respectively, released MN with similar profiles: at 12 h 45.58–50.31% and at 24 h 63.56–73.31% of the 40 mg—the calculated dose of the film samples. FII film released MN at a lower rate than FIII, the releasing capacity expressed by the area under the curve (AUC) having quite close values after 24 h: FII—1.816 mg·h·mL^−1^, while FIII—2.094 mg·h·mL^−1^.

The comparative analysis of release profiles (mg·cm^2^ vs. h) linearized by simple regression of the segments corresponding to the first 12 h (Figure 3b) shows that the deviation from zero is significant for both lines (*p* < 0.0001) while the deviation from linearity is not significant (FII—*p* = 0.6429, FIII—*p* = 0.5000), and there are no significant differences between the two curves, neither for the slopes (*p* = 0.37) nor for the intercepts as measure of elevations (*p* = 0.74). Thus, there was the possibility of calculating the average value of the slopes (pooled slope = 0.61), and also of the intercepts (pooled intercept = 0.5305). The R square coefficient indicates a good determination (of 96–98%), with the release/diffusion rate of 0.59–0.64 mg·cm^−2^·h^−1^ MN through the synthetic membrane.

Although a wide range of oral pharmaceutical drugs use as polymer HPCM with high molecular mass, in case of the studied miconazole nitrate films this excipient makes the release of the antifungal more difficult, probably due to MN’s low solubility in water. The use of HEC as film forming polymer behaves better than HPMC in terms of the release/diffusion processes. Thus, a concentration of 2% HEC determines a film matrix which releases up to 70% miconazole nitrate, unlike the matrix film prepared with 3% HEC which releases only up to 60%. Practically, when the concentration level increased by 1% HEC, the amount of MN released decreased; this makes us conclude that HEC should not exceed 2% in the fluid casted phase for optimal release of MN from the HEC-based matrix films.

The jellification capacity of HEC—a non-toxic thickening agent—responded better to the requirements of a MN dermal or mucosal film, unlike HPMC which, although having the advantage of not dehydrating the skin or the mucosa, decreases the MN release processes significantly, probably due to its great capacity of absorbing and retaining the water.

Regarding the published data, Saudagar R.B. (2017) obtained about a 82–99% MN release (24 h) when studying film formulations prepared with Eudragit RS-PO/hydroxypropyl cellulose (HPC), using egg membrane for the in vitro study [39]. Ofokansi K.C. (2015) obtained values of in vitro permeation flux of about 5 µg·cm^−2^·h^−1^ MN permeated through rat skin with, using in formulations mixtures of film forming polymers Eudragit RS-100/hydroxypropyl methylcellulose (HPMC) [40]. However, the different experimental conditions used in these studies do not allow comparisons in terms of delivery efficiency.

#### 2.3.2. Analysis of In Vitro Release Profiles

In order to determine the kinetics and mechanism of MN release, the release profiles of the two HEC films (FII—prepared with 2% and FIII—with 3% polymer) were fitted using the first order kinetic equation in four variants: the simple equation; the equation considering the latency time (T_lag_); the equation considering the maximum fraction that can be released using the gradient created by the initial dose of MN in the film (F_max_); and the equation that considers both T_lag_ and F_max_ parameters, respectively (Table 3).

The analysis of the calculated data indicates for both studied films (FII and FIII) that the highest value of R_sqr_adj_ (the adjusted coefficient of determination) was obtained by fitting the release profiles—% (m/m) vs. time (h) with the model no. 4—first order kinetic with T_lag_ and F_max_, this proving to be the closest model to the studied time-dependant curves (of the four models used) [41]. The kinetic parameters k_1_, T_lag_ and F_max_ quantify the release differences and allow the comparisons of the kinetic release processes of MN from the two HEC films matrix, which differ only by the concentration of polymers used in the film preparation: 2% for FIII and 3% for FII. The results show that the additional use of 1% HEC in the film composition generates the modification of k_1_ (the constant of the release process kinetic) by 0.06^−1^ (as the difference between k_1_-FII and k_1_-FIII), accompanied by a tripling (from 11.57 to 35.99%) of the amount of MN that could be released from a film of 2.54 cm^2^ × (0.30 − 0.23) mm.

## 3. Materials and Methods

### 3.1. Preparation of MN-Dermal Films

#### 3.1.1. Materials

Miconazole nitrate (MN) was purchased from Sigma Aldrich Inc. (Darmstadt, Germany). Two types of cellulose ether polymers were used as matrix formers in ultrapure water (Direct-Q water purification system Merck Millipore, Darmstadt, Germany): Natrosol^TM^ 250M (hydroxyethyl cellulose-HEC, viscosity of 4500–6500 mPa·s) from Ashland (Düsseldorf, Germany), Hypromellose 90SH (hydroxypropyl methylcellulose-HPMC, viscosity of 15,000 mPa·s) from Shin-Etsu Chemical Co., Ltd. (Tokyo, Japan). Auxiliary substances: propylene glycol (PG) from Scharlau Chemie (Barcelona, Spain), polyethylene glycol 400 (PEG_400_) and polysorbate 20 (PSB_20_) from Sigma Aldrich Inc. (Hamburg, Germany), ethanol from Stireco LTH (Buzau, Romania).

#### 3.1.2. Films Preparation Technique

MN was dissolved in alcohol by stirring (500 rpm) for 5 min. PEG_400_, distilled water and in the end the film forming polymer (HPMC or HEC) are successively added under constant stirring, continuing the stirring for another 25 min after dispersion preparation, avoiding evaporation. The air was then eliminated from the structured gel by maintaining the fluid phase for 25 min in the ultrasound bath. The resulting composition was then poured onto circular plates (diameter of 9.8 cm) and left to dry at 40 °C (24 h) [42]. The films obtained were used in the study after 48 h of preservation at 20 °C, protected from light and moisture.

### 3.2. Evaluation of Physicochemical and Mechanical Parameters

#### 3.2.1. Measurement of Physical Parameters

The appearance of the dermal films was visually analysed. The mass of each film was established by weighing. The thickness (Tick) was determined using a micrometre, by measuring it at five different points [43].

#### 3.2.2. Mechanical Resistance Test

The film sample of 3.5 cm long (L_i_) and 1.5 cm wide (W) was fixed at one of the ends to a fixed support, while at the other end were placed heavier weights—m (g) until the film broke (M_f_), measuring each time the length of the film—L_f_ (cm).

The film elongation-E was calculated as percentage of the initial length, using the Equation (1):

(1) $$E\%=\left(L_{f}-L_{i}\right)\times 100/L_{i}$$

The tensile strength-T_s_ (N·mm^−2^) was calculated using the Equation (2):

(2) $$T_{s}=M_{f}\left(\mathrm{kg}\right)\times 9.81\left(N\cdot {\mathrm{kg}}^{-1}\right)/S\left({\mathrm{mm}}^{2}\right),$$

where S = W (mm)·Tick (mm) [43].

#### 3.2.3. Stickiness (Adhesion) Test (S_t_)

The weight—m (g) which, with the use of a certain lever, generated a vertical ascendant force which lead to the separation of a smooth plate from the surface—S (cm^2^) of the film sample, onto which it was previously pressed and attached, was determined for each film sample.

The calculation considered the Equation (3):

(3) $$S_{t}\left(N\cdot {\mathrm{cm}}^{-2}\right)=M\left(\mathrm{kg}\right)\times 9.81\left(N\cdot {\mathrm{kg}}^{-1}\right)/S\left({\mathrm{cm}}^{2}\right)$$

#### 3.2.4. Water Vapour Absorption Test (A_w_)

The film sample weighing m_i_ (g) was maintained in a sealed compartment, in the presence of KCL_2_ saturated solution (80% relative humidity-RH), up to a constant weight—m_f_ (g).

The calculation used the Equation (4):

(4) $$A_{w}\left(\%\right)=\left(m_{f}-m_{i}\right)\times 100/m_{i}$$

#### 3.2.5. Water Vapour Loss by Desiccation Test (L_w_)

The film sample weighing m_i_ (g) was maintained in a sealed compartment, in the presence of CaCl_2_ anhydrous (0% RH), up to a constant weight—m_f_ (g). The calculation used the Equation (5):

(5) $$L_{w}\left(\%\right)=\left(m_{f}-m_{i}\right)\times 100/m_{i}$$

#### 3.2.6. Water Vapour Permeability Test (P_w_)

The film sample with the determined surface of s (cm^2^) was attached so as to close a glass containing CaCl_2_ anhydrous (0% RH), resulting an ensemble with the mass—m_i_ (g), which afterwards was kept in a sealed compartment in the presence of KCl_2_ saturated solution (80% RH), up to the constant mass—m_f_ (g). The calculations considered the Equation (6):

(6) $$P_{w}\left(g\cdot {\mathrm{cm}}^{-2}\cdot h^{-1}\right)=\left(m_{f}-m_{i}\right)\times 100/s\;\left({\mathrm{cm}}^{2}\right)/t\left(h\right)$$

### 3.3. Evaluation of Dermal films In Vitro Availability

#### 3.3.1. Determination of In Vitro Release Profiles

Franz cells of 14 mL capacity and 1.8 cm diameter were used. The diffusion membrane (Teknokroma Ø 25 mm × 0.45 μm) was prepared before each determination by maintaining it in a phosphate buffering solution pH 7.4.

The samples analysed consisted of disk shaped films with diameter of 1.8 cm (surface of 2.54 cm^2^). The media consisted in a buffer solution pH 7.4 to which was added 0.045% sodium lauryl sulphate.

During the determination: the diffusion cells were maintained at 32 ± 0.5 °C, by heating in recirculated water, with oscillating (electromagnetic) stirring. The samples taken (5 mL) were replaced each time with fresh media, and the MN content was assessed by ultraviolet (UV)-spectrophotometric method, at the wave length of λ = 273 nm (Spectrometer HP 8451A UV-VIS, Hewlett Packard, Palo Alto, CA, USA).

The calculations considered Equation (7):

(7) $$\mathrm{MN}=m\left(n-1\right)+14C_{\mathrm{pn}}-\;9C_{p}\left(n-1\right),$$

where MN (mg)—mass of miconazole nitrate released at moment *n* (collection *n*), C_pn_-the concentration of miconazole nitrate expressed in mg/mL for sample collected at moment n, 14-the volume of Franz cell expressed in mL, 9-the volume expressed in mL of the acceptor media that remained in Franz cell after the collection of the 5 mL sample [44,45].

#### 3.3.2. The Release Profile

The release profile was determined by plotting the cumulative amount of MN released (% m/m of calculated dose of film sample, or mg·mL^−1^ of the acceptor media, or mg·cm^−2^ of the sample surface) vs. time (h).

#### 3.3.3. Kinetic of Release Modelling

Kinetic of release modelling [46], was performed with the DDSolver Add-Ins software, using the kinetic equations: first-order, first-order with T_lag_, first-order with F_max_, first-order with T_lag_ and F_max_. R^2^ adjusted coefficient was used as goodness of fit parameter in order to discriminate the most appropriate model: the higher the value, the better the model fitting.

### 3.4. Statistical Analysis

GraphPad Prism 6 software was used, running the Pearson correlation (r) test and linear regression test, followed by the runs test; with the statistical significant difference set at *p* < 0.05, for a CI of 95% [46].

## 4. Conclusions

Three compositions containing 1% HPMC 15,000, 2% or 3% HEC 250 M were formulated and processed by casting solvent evaporation technique, in order to develop new antifungal dermal films in the form of a polymeric adhesive matrix containing MN 10 mg/cm^2^ or 40 mg/cm^2^.

The behaviour towards moisture vapour indicates that the MN-HPMC film has no proper properties in terms of mechanical resistance, elongation or ability to release, in a prolonged way, the MN over a period of 24 h. This formulation could eventually be improved through extending the drying period or/and by increasing the amount of plasticizer (PEG_400_).

The MN-HEC films are elastic, resistant to stretching and have the ability to release more than 60% of their MN content in vitro at pH 7.4, especially if less than 3% HEC is used in the preparation process. Considering the difficultly of the MN dissolution (release) process due to its low solubility in aqueous media, the release of 60–70% from these new dermal films can be considered a very good percentage. The most appropriate composition for MN dermal film preparation is represented by FIII—obtained with 2% HEC. Its breakage strength and high elasticity creates favorable prerequisites for the management of operations which could generate mechanical stresses, for example, during the technological process of film preparation, or for maintaining the integrity of a product during its administration onto the skin or mucosa. Also, elasticity is a property that can be quantified by mathematical (rheological) functions, so it could be used by studying its correlation with MN release ability, and predicting certain qualities by the design of the experiment.

## Figures and Tables

**Table 1:** Composition of the liquid phases casted to obtain the miconazole nitrate films.

Ingredient	Code	Formula/Quantity (% m/m)			Function
		FI	FII	FIII	
Miconazole nitrate	MN	1.25	5.00	5.00	Active pharmaceutical ingredient
Hydroxyethyl cellulose 250 M	HEC	-	3.00	2.00	Film forming agent, non-ionic, water highly viscous soluble polymer
Polyethylene glycol 400	PEG_400_	-	1.00	1.00	Permeation enhancer, solubilizer, plasticizer
Hydroxypropyl methylcellulose 15,000	HPMC	1.00	-	-	Film forming agent, non-ionic, water highly viscous soluble polymer, water retention agent
Propylene glycol	PG	10.00	-	-	Humectant agent, solubilizer, plasticizer
Polysorbate 20	PSB_20_	1.00	-	-	Surfactant, non-ionic, oil in water emulsifier, permeation enhancer, solubilizer
Ethanol	-	30.00	10.00	10.00	Co-solvent
Ultrapure water	-	56.75	81.00	82.00	Solvent
Total		100	100	100	

**Table 2:** Physical characteristics of the obtained miconazole nitrate films.

Parameter	Dermal Film Formula		
	FI	FII	FIII
Appearance	Translucent film	Opaque film	Opaque film
Surface	Slightly rough	Shiny smooth	Shiny smooth
Thickness (mm ± SD ^1^)	0.20 ± 0.009	0.30 ± 0.004	0.23 ± 0.007
MN theoretical content			
mg in 63.585 cm^2^ film:	250	1000	1000
mg in 2.54 cm^2^ film:	10	40	40
mg·cm^−2^:	3.931	15.726	15.726

^1^ SD = standard deviation (*n* = 3).

**Table 3:** The kinetic parameters of release profiles fitted with first-order kinetic equations.

Model	Kinetic Function/Equation ^1^ [41]	Parameter ^2^	FII	FIII
1	First-order $F=100\times \left[1-\mathrm{Exp}\left(-k_{1}\times t\right)\right]$	$k_{1}$	0.0071	0.0072
		$R_{\mathrm{sqr}\_\mathrm{adj}}$	0.9361	0.9750
2	First-order with $T_{\mathrm{lag}}$ $F=100\times \left\{1-\mathrm{Exp}\left[-k_{1}\times \left(t-T_{\mathrm{lag}}\right)\right]\right\}$	$k_{1}$	0.0516	0.0543
		$T_{\mathrm{lag}}$	−0.3832	−0.0765
		$R_{\mathrm{sqr}\_\mathrm{adj}}$	0.9848	0.9774
3	First-order with $F_{\max}$ $F=F_{\max}\times \left[1-\mathrm{Exp}\left(-k_{1}\times t\right)\right]$	$k_{1}$	0.0903	0.0374
		$F_{\max}$	10.8379	21.6643
		$R_{\mathrm{sqr}\_\mathrm{adj}}$	0.9872	0.9794
4	First-order with $T_{\mathrm{lag}}$ and $F_{\max}$ $F=F_{\max}\times \left\{1-\mathrm{Exp}\left[-k_{1}\times \left(t-T_{\mathrm{lag}}\right)\right]\right\}$	$k_{1}$	0.0799	0.0200
		$T_{\mathrm{lag}}$	−0.1667	−0.3614
		$F_{\max}$	11.5726	35.9946
		$R_{\mathrm{sqr}\_\mathrm{adj}}$	0.9864	0.9795

^1^ F—the fraction (%) of drug released in time t (h); F_max_—the maximum fraction (%) of the drug released at infinite time; ^2^ k_1_—the first-order release constant (h^−1^); T_lag_—the lag time prior to drug release; R_sqr_adj_—the adjusted coefficient of determination.
